# Acute Kidney Injury Induces Innate Immune Response and Neutrophil Activation in the Lung

**DOI:** 10.3389/fmed.2020.565010

**Published:** 2020-11-20

**Authors:** Akinori Maeda, Naoki Hayase, Kent Doi

**Affiliations:** Department of Acute Medicine, The University of Tokyo Hospital, Tokyo, Japan

**Keywords:** neutrophil, IL-6, high mobility group box 1, toll-like receptor, neutrophil extracellular trap

## Abstract

Complication in acute kidney injury (AKI) is significantly associated with developing acute respiratory failure (ARF), while ARF is one of the most important risks for AKI. These data suggest AKI and ARF may synergistically worsen the outcomes of critically ill patients and these organ injuries may not occur independently. Organ crosstalk between the kidney and the lung has been investigated by using animal models so far. This review will focus on innate immune response and neutrophil activation among the mechanisms that contribute to this organ crosstalk. AKI increased the blood level of an inflammatory mediator in high-mobility group box 1, which induces an innate immune reaction via toll-like receptor 4. The remarkable infiltration of neutrophils to the lung was observed in animal AKI models. IL-6 and IL-8 have been demonstrated to contribute to pulmonary neutrophil activation in AKI. In addition, the formation of a neutrophil extracellular trap was also observed in the lung after the exposure of renal ischemia reperfusion in the animal model. Further investigation is necessary to determine whether targeting innate immune response and neutrophil activation will be useful for developing new therapeutics that could improve multiple organ failure in critically ill patients.

## Introduction

Acute kidney injury (AKI) is a common complication in critically ill patients treated in the intensive care unit (ICU). Reportedly, AKI occurs in ~30–60% of patients admitted to the ICU and is associated with significantly poor outcomes including morbidity and mortality (1). ICU patients with AKI will be complicated by a broad range of other organ injuries including heart failure, respiratory failure, liver injury, and immunological abnormalities. Many animal studies demonstrated that AKI induces cardiac, lung, liver, brain, and splenic injury (2). Therefore, AKI should be considered as a systemic disease that can have significant impacts on distant organs.

Though the primary roles are different, lung and kidney functions are intimately related regarding the maintenance of homeostasis. The blood acid-base balance is regulated by carboxy dioxide excretion by the lung and bicarbonate reabsorption by the kidney. A compensative mechanism will activate when one organ fails to keep these levels within a normal range by the other organ. Thus, respiratory complication in AKI patients is particularly important. Clinical studies revealed AKI patients were twice as likely to require invasive mechanical ventilation (3). On the other hand, acute respiratory failure (ARF)/acute respiratory distress syndrome (ARDS) patients, especially those who require invasive mechanical ventilation, are at high risk for developing AKI (4, 5).

In this review, we provide a brief overview regarding (1) the clinical data on AKI and ARF/ARDS and (2) the potential mechanisms by which AKI leads to lung injury. Recently identified mechanisms of the innate immune system including toll-like receptors (TLRs) and damage-associated molecular patterns (DAMPs) were evaluated in the kidney-lung crosstalk using animal models. Neutrophil extracellular traps (NETs) was identified as another player in the innate immune response. NETs were also demonstrated to contribute to AKI-induced lung injury.

## Clinically Reported Associations Between AKI and ARF/ARDS

Many clinical studies suggested associations between AKI and ARF/ARDS in critically ill patients. AKI often occurs in ARF/ARDS patients, while ARF/ARDS is often observed in AKI patients. First, clinical data about AKI complication in ARF/ARDS are described below. The secondary analysis of a randomized control trial conducted by the ARDS network reported AKI occurred in ~25% of ARDS patients. The mortality rate of AKI patients was significantly higher than the non-AKI patients (6). Another study reported that AKI was observed in ~35% of hospitalized patients with community-acquired pneumonia. AKI was significantly associated with the requirement of mechanical ventilation (MV) and mortality (4). A meta-analysis including 31 studies reported a significantly high AKI occurrence when the patients were mechanically ventilated (5). These data suggest that AKI is a common complication and a risk factor for poor outcomes of ARF/ARDS.

Second, ARF/ARDS complication in AKI is described below. Severe ARF requiring MV was observed in more than 70% of AKI patients in a multinational, multicenter prospective observational study. MV requirement was significantly associated with higher mortality (7). Dialysis-requiring AKI (AKI-D) is the most severe form of AKI (defined as AKI stage 3 by the KDIGO criteria). A small retrospective study reported that 44% of AKI-D patients treated in an ICU developed ARDS (8). Therefore, ARF/ARDS is a common complication and a risk factor for poor outcomes of AKI.

Third, AKI and ARF/ARDS are organ failures that can be simultaneously complicated in a critically ill patient. An observational study conducted in 18 French ICUs reported ARDS occurrence as 23%, while AKI occurrence was at 31%. Hospital mortality was 14% in this population and it increased to 42% in patients with both AKI and ARDS (9). In a large cohort of 200,000 admissions for subarachnoid hemorrhage (SAH) from the Nationwide Inpatient Sample database, the incidence of ARDS in SAH patients was ~35% and renal dysfunction predicted ARDS development (10). A nationwide study including ~7 million patients with acute ischemic stroke showed AKI patients had a significantly higher risk of requiring MV than non-AKI (11). As described above, both AKI and ARF/ARDS are common organ failures in critically ill patients with other etiologies such as sepsis and the coexistence of these issues will exacerbate the outcomes. However, it should be noted that these clinical observational studies do not indicate causal relationships but only associations between these two types of organ failures.

## Possible Mechanism of Organ Crosstalk Between the Kidney and the Lung

The pathophysiological mechanisms of respiratory failure in patients with AKI can be categorized into inflammatory and non-inflammatory mechanisms (Table 1). Non-inflammatory mechanisms include fluid overload and cardiac dysfunction. Rapid progression of lung edema and fluid overload, which do not respond to diuretics, are frequently observed in AKI patients. Although inflammatory mechanisms are not well-recognized in basic clinical research, using animal models demonstrated that increased inflammatory mediators in AKI play a crucial role in lung injury.

**Table 1:** Pathophysiological mechanisms of respiratory failure in AKI.

Non-inflammatory	Fluid overload
	Cardiac dysfunction
Inflammatory	Increased cytokine levels (tumor necrosis factor-α, IL-6, and IL-8)
	Neutrophil activation
	Pulmonary endothelial apoptosis
	Oxidative stress

Rabb and colleagues first demonstrated increased inflammatory gene expressions in the lung after exposure of renal ischemia reperfusion injury by using microarray analysis (12). Faubel et al. (13) demonstrated the role of IL-6 in AKI-induced lung injury with IL-6 knockout mouse experiments. The reduced clearance of inflammatory mediators including IL-6 in AKI seemed to contribute to lung injury. Furthermore, they reported that IL-6-induced expression of the chemokine CXCL1 in the lung was responsible for recruiting neutrophil into the lung induced by AKI (14). These findings suggest that circulating IL-6 is a pathogenic mediator of lung injury in AKI. IL-10, an anti-inflammatory cytokine, was shown to be produced by spleen CD4-positive T cells in response to IL-6 in AKI. The induction of IL-10 expression by IL-6 appeared to contribute to the suppression of lung injury induced by AKI as a counterbalance (15). Other mediators and inflammatory cells were also reported as possible contributors to lung injury induced by AKI (Table 2).

**Table 2:** Mediators of lung injury in AKI.

IL-6	Serum IL-6 can be used as a predictor of AKI
	Serum IL-6 level elevates in the very early stage of AKI
	Higher level of serum IL-6 is associated with the prolonged mechanical ventilation in patients with AKI
	IL-6 antibody-treated mice exhibit less lung inflammation and fewer capillary leaks
	IL-6 deficient mice exhibit less lung inflammation and fewer capillary leaks
	Intravenous IL-6 injection to IL-6 deficient mice restores lung inflammation
	Circulating IL-6 causes lung inflammation in AKI
IL-8	Serum IL-8 levels elevate in the very early stage of AKI
	Higher level of serum IL-8 is associated with the prolonged mechanical ventilation in patients with AKI
	Higher level of serum IL-8 is a predictor of increased mortality in patients with AKI
	IL-8 antibody-treated mice exhibit less lung injury
	IL-8 deficient mice exhibit less lung injury
TNFR1 + caspase-3- mediated apoptosis	Circulating TNF and caspase-3 increase endothelial apoptosis and lead to non-cardiogenic pulmonary edema
	Pan-caspase inhibition reduces pulmonary edema after AKI
	TNF inhibition reduces apoptosis and pulmonary edema after AKI
	TNFR1 deficient mice exhibit less lung caspase-3 and lung apoptosis after AKI
T cells	T cells participate in lung apoptosis via caspase-3 and lead to non-cardiogenic pulmonary edema
	T cells are recruited to the lung within 24 h after AKI
	T cell deficient mice exhibit less caspase-3 and less pulmonary edema

*TNFR1, tumor necrosis factor receptor 1*.

Although these studies suggested that the induction of an inflammatory reaction by AKI is involved in lung injury, other studies demonstrated AKI suppressed inflammatory reactions and subsequently worsened pulmonary infection. Neutrophil function against pneumonia was evaluated in the model of bacterial pneumonia in mice complicated with AKI by folic acid administration or glycerol injection (rhabdomyolysis) (16). Neutrophil migration was rather weakened and the pneumonia was exacerbated in AKI animals. Another study reported that the neutrophil migration ability at microcirculation of the cremaster muscle was decreased in AKI mice (17). AKI-induced neutrophil dysfunction might be caused by an adipokine resistin (18). Resistin is known as a uremic toxin and pro-inflammatory cytokine secreted by monocytes, neutrophils, and epithelial cells (19). It should be noted septic patients in the ICU showed higher blood levels of resistin than non-septic patients (20).

Taken together, increased humoral mediators by reduced clearance and increased expression in AKI seem to contribute to lung injury. On the other hand, few studies revealed the mechanisms of the opposite direction of crosstalk, i.e., ARF/ARDS-induced kidney injury. Slutsky et al. (21) reported increased epithelial cell apoptosis in the kidney in a rabbit ARDS model followed by mechanical ventilation with injurious ventilatory strategies (high tidal volume and high PEEP). An infiltration of lymphocytes was seen in the renal cortex in a pig ARDS model with mechanical ventilation (22). On the other hand, another study found no renal structural and functional alterations in a canine ARDS model when hemodynamics and arterial blood gas tensions are carefully controlled (23), indicating the possible role of a non-inflammatory mechanism for kidney injury in ARDS. Further investigation is necessary for whether inflammatory mediators play any role in ARF/ARDS-induced kidney injury.

## Innate Immune Response and Neutrophil Activation

Neutrophil infiltration is a major finding in lung injury induced by AKI. A remarkable neutrophil infiltration in the lung together with increased neutrophil elastase (NE) activity in blood and lung tissues were observed in a mouse AKI model and specific NE inhibitor reduced lung injury (24). Toll-like receptors (TLRs) are pattern recognition receptors that recognize pathogen-associated molecular patterns (PAMPs) and damage-associated molecular patterns (DAMPs). TLRs play an important role in innate immunity, and are involved not only in the defense against infection but also in various pathological conditions. An inflammatory mediator, high-mobility group protein B1 (HMGB1) is one of the DAMP TLR4 ligands. We revealed that TLR4 loss-of-function mutant mice (C3H/HeJ) were resistant to lung injury caused by AKI (25). Although blood levels of HMGB1 increased in AKI regardless of TLR4 mutation, anti-HMGB1 neutralizing antibody treatment reduced lung injury only in the wild-type mice, indicating the organ protecting effect was mediated by the HMGB1-TLR4 pathway.

Extracellular histones induce cytotoxicity as DAMPs trigger the inflammatory cascade via toll-like receptors. Extracellular histones are released from neutrophils during the formation of neutrophil extracellular traps (NETs), that comprise DNA studded with histones and proteases, including neutrophil elastases (26). Extracellular histones and NETs were reported to associate with the pathogenesis of acute lung injury in an animal AKI model. Nakazawa et al. (27) showed that the induction of necrosis in renal tubular epithelial cells *in vitro* caused by hypoxia stimulated neutrophils to form NETs by extracellularly released histones. In a mouse intestinal ischemia-reperfusion model, extracellular histone accumulation and NETs formation were observed in the liver rather than the intestine (28). Extracellular histones derived from the intestinal tract were considered to transported to the liver via the portal system. In the mouse renal ischemia-reperfusion injury model, elevated extracellular histones in blood and NETs formation in the lung was observed (27). Human recombinant thrombomodulin (rTM) reportedly trap extracellular histones *in vitro*. Although no renal protection by rTM was observed in the renal ischemia-reperfusion model, significant improvement of lung injury together with NTEs accumulation induced by AKI was observed in rTM-treated animals (29).

These findings above suggest that extracellular histones and NETs formation might be responsible for AKI-induced lung injury, although other pathways of inflammatory mediators such as IL-6 also contribute to AKI-induced lung injury. For the development of new therapeutics against organ crosstalk between the kidney and lung, targeting multiple pathways will be necessary. In addition, extracellular histones and NETs formation have not been sufficiently evaluated in human ARDS (30) and no clinical study examined this pathway in terms of lung-kidney crosstalk. Further evaluation of these pathways in a clinical setting is absolutely required.

## Possible Organ Crosstalk Between the Kidney and the Lung in COVID-19

The novel coronavirus disease (COVID-19) caused by severe acute respiratory syndrome coronavirus 2 (SARS-CoV-2) was first reported in Wuhan in December 2019, which has spread around the world. SARS-CoV-2 dominantly affects the respiratory system and recent clinical studies reported AKI as a significant comorbidity in COVID-19 (31). The cell surface protein, angiotensin-converting enzyme 2 (ACE-2), which is used by the virus as an entry receptor, is abundantly expressed not only in lung epithelial cells but in renal tubular epithelial cells (32), indicating SARS-CoV-2 could directly infect the kidneys. We recently observed that the urinary neutrophil gelatinase-associated lipocalin (NGAL) level at ICU admission was significantly elevated among patients who developed AKI. Furthermore, urinary NGAL was correlated with length of mechanical ventilation use (33). Because urinary NGAL is expected to reflect renal tubular epithelial cell damage, elevated NGAL might reflect the burden of viral insult of renal damage directly and lung injury indirectly.

## Discussion

Multiple organ failure frequently observed in critically ill patients was previously considered as the “sum” of each organ failure. The sequential organ failure assessment (SOFA) score, which is widely used for evaluating the severity of ICU patients, is calculated by summing up each organ injury score. No consideration for organ crosstalk can be found in these scoring systems. Several clinical studies demonstrated possible organ crosstalk in ICU patients by applying network analysis (34, 35). As described above, basic research revealed several different pathways that contributed to organ crosstalk related to AKI (36). Unfortunately, no clinical evidence is available for organ crosstalk demonstrated by animal experiments. Future studies that could clarify the significance of organ crosstalk in the clinical setting is necessary to develop a novel therapeutic strategy that targets currently unrecognized organ crosstalk in critically ill patients.

This review covers kidney-lung crosstalk mostly regarding lung injury caused by AKI. The opposite pathological mechanism in which ARF/ARDS causes AKI should also be considered. As described above, only a limited number of basic studies reported possible mechanisms in animal experiments. In clinical studies, ARF/ARDS caused a systemic release of pro-inflammatory mediators (plasminogen activator inhibitor-1, IL-6, and soluble TNF receptors-1 and 2), which could induce or worsen AKI (6). AKI can be caused by a reduction in renal blood flow caused by hypoxemia or hypercapnia, and a decreased glomerular filtration rate due to the elevation of central venous pressure (renal congestion). In patients with ARF/ARDS, these mechanisms could initiate or aggravate AKI and cause those patients to fall into a vicious cycle (37).

Mechanical ventilation-induced lung injury has been investigated so far. Low tidal ventilation strategy against ARDS is recommended based on evidence obtained by both basic and clinical studies (38). Does MV have any significant impacts on the kidney injury? Excessive tidal volume leads to proinflammatory mediator release. High positive end-expiratory pressure can cause elevated intrathoracic pressure and systemic venous pressure, leading to a reduced net glomerular filtration rate. Lung protective ventilation based on the volume-pressure curve can achieve lower levels of TNF-α, IL-1b, IL-6, and IL-8 in bronchoalveolar lavage fluid with a lower incidence of AKI in the clinical setting (39).

## Author Contributions

AM, NH, and KD contributed to the writing of the manuscript and approved the final version of the manuscript.

## Conflict of Interest

The authors declare that the research was conducted in the absence of any commercial or financial relationships that could be construed as a potential conflict of interest.
